# Effect of Nitrophos Fertilizer on Pollinator Dynamics and Onion Seed Yield

**DOI:** 10.3390/biology14020119

**Published:** 2025-01-23

**Authors:** Syeda Fatima Bukhari, Mudssar Ali, Fawad Zafar Ahmad Khan, Raimondas Mozūratis

**Affiliations:** 1 Institute of Plant Protection, Muhammad Nawaz Shareef University of Agriculture Multan, Multan 60000, Pakistan; fatimabukhari45@gmail.com (S.F.B.); fawad.zafar@mnsuam.edu.pk (F.Z.A.K.); 2 Department of Outreach and Continuing Education, Muhammad Nawaz Shareef University of Agriculture Multan, Multan 60000, Pakistan; 3 Laboratory of Chemical and Behavioural Ecology, Institute of Ecology, Nature Research Centre, LT-08412 Vilnius, Lithuania; 4 Department of Zoology, Stockholm University, SE-10691 Stockholm, Sweden

**Keywords:** pollination ecology, honey bees, syrphid flies, umbel weight, nutrient–pollination interaction, sustainable agriculture

## Abstract

The yield of flowering crops depends on multiple factors, with pollination and the availability of nutrients being particularly crucial. However, fewer studies have explored the combined effects of nutrients and insect-mediated pollination on onions (*Allium cepa* L.). Here, we studied how different levels of insect pollination (0%, 25%, 50%, and 100%) and nitrophos fertilizer application (188, 375, and 750 kg/hectare) affect onion seed yield. We found that pollination benefits to yield were maximum at intermediate levels of nitrophos availability. A higher pollinator abundance was recorded at a high nitrophos level, followed by intermediate and low levels. Syrphid flies and honey bees were the most abundant insect pollinators. The abundance of honey bees was higher at a low nitrophos level, while syrphid flies were more abundant at a higher nitrophos level. Moreover, the umbel weight and seed number were higher at the intermediate fertilizer level. Also, single visits by insect pollinators were observed, and the highest umbel weight was found at the intermediate fertilizer level with a single visit from *A. florea*. This finding provides evidence that resource and pollination availability have interactive, nonlinear effects on seed production. Our results also support ecological intensification as a reliable strategy for sustainable agro-ecosystem management.

## Simple Summary

The seed yield of flowering crops is influenced by nutrient availability and pollination. However, the combined effects of these factors have not been extensively studied. This research investigated the impact of varying insect pollination levels (0%, 25%, 50%, and 100%) and nitrophos fertilizer doses (188, 375, and 750 kg/hectare) on onion seed production. The results demonstrated that onion seed yield increased with pollination at moderate fertilizer levels. Pollinator abundance varied with fertilizer levels; honey bees were more prevalent at lower fertilizer levels, while syrphid flies were more abundant at higher levels. The umbel weight and number of seeds per umbel were highest with the intermediate fertilizer treatment. Additionally, our findings highlighted the single-visit effectiveness of the *Apis florea* bee. The data revealed a complex interplay between fertilizer treatment and pollination services, underscoring the role of insect pollination in enhancing onion seed production.

## 1. Introduction

Promoting insect pollination and improving crop diversity through non-conventional farming practices are essential to enhance food security and sustainability [1]. Even in self-compatible crops, pollination is necessary for maximum seed and food production [2]. Crops have varying pollination needs, and many depend on insect pollinators for pollen transfer, ensuring successful fertilization and fruit and seed production [3,4,5]. Many crops depend partially or entirely on insect pollination services.

After tomatoes, onions are the second most widely consumed vegetable globally and have the most extended history of cultivation [6]. They are consumed both raw in salads and in processed form [7]. Onion flowers are protandrous, i.e., releasing pollen before the stigma becomes receptive; therefore, cross-pollination relies mainly on biotic factors. The anther produces sticky, wet pollen grains, and, due to this factor, insect pollination contributes more than wind pollination [8]. A few studies demonstrated that effective pollination by insects leads to significantly higher seed set and yield (44 and 6 times greater, respectively) compared to self-pollinated plants [9,10]. Another study found that a lack of insect pollinators can result in a 5–14% reduction in seed quantity and a decline in seed quality [11,12].

A strong linkage between crop management practices and insect-mediated pollination has been reported [13,14]. Studies have shown that water and nutrient levels affect pollination success [13,15]. Moreover, these factors also influence pollinator health and behavior due to changes in floral resource quality and availability. For example, the maximum yield in okra, *Abelmoschus esculentus* (L.) (Moench) (Malvaceae), depends on optimal soil fertilization coupled with adequate pollinator visitation [1]. A study on common beans, *Phaseolus vulgaris* L. (Fabaceae), also showed that low nitrogen levels led to a higher abundance of insect pollinators [16]. Similar results have been reported in oil-seed rape (*Brassica napus* L. var. *oleifera*) (Brassicaceae), where low nitrogen application led to increased insect pollination [17]. Another study on sunflowers (*Helianthus annuus* L.) (Asteraceae) reported higher pollinator visitation and yield at intermediate nitrogen levels [18]. Moreover, ornamental flower Scarlet gilia *Ipomopsis aggregata* (Pursh) V.E.Grant (Polemoniaceae) and Lewis flax *Linum lewisii* Pursh (Linaceae) showed no changes in reproductive success due to fertilizer application [19].

Nutrients indirectly affect pollen quantity and quality [20], flower production, pollination, pollinator attraction, and seed setting [19]. Soil-available nutrients (nitrogen, phosphorus, and potassium) have an important influence on floral traits and pollinator attraction [21]. The insect visitation rate has been reported to correlate to nectar amino acids, pollen fatty acids, and amino acids [22]. Nitrogen and phosphorus are key nutrients that enhance crop productivity [23]. However, their use also negatively affects the environment by increasing water and soil eutrophication and greenhouse gas emissions [24,25]. In insects, bees are the most important pollinator group for plant reproduction [26]. Wild bees are essential for improving the productivity of pollinator-dependent crops [27], but their abundance and diversity are negatively affected by agricultural intensification [28]. The combined impact of pollination services and fertilizer application on onion yield has not been documented in the literature.

The current study aimed to identify the effect of soil fertilization and pollinator visitation on onion yield. To test the hypothesis that insect pollination services could be maximized at specific levels of nitrophos application, we created various insect-mediated pollination gradients along with the nitrophos application levels and assessed the onion seed yield.

## 2. Materials and Methods

### 2.1. Plant Selection

We selected the red phulkari variety of onion (*A. cepa* L.) because it has been cultivated on a large scale in Pakistan. Onions are biennial herbaceous plants grown for their edible bulbous base. Their umbels typically contain 50–2000 florets enclosed in 2–3 white-colored spathes. Being protandrous, onions require cross-pollination, and insect-mediated pollination is important in determining the final seed yield [29].

### 2.2. Experimental Site and Design

This study was conducted from November 2019 to April 2020 at the experimental farm of Muhammad Nawaz Shareef University of Agriculture, Multan, Pakistan. The Multan district has been categorized as a subtropical desert due to its hot summers and chilly winters, with temperatures ranging from 38 to 50 °C at maximum and from 8 to 12 °C at minimum. During summer, the average monthly rainfall is around 18 mm.

We arranged nine plots into three blocks using a randomized complete block design, with each block containing three plots. Each plot measured 15.24 m in length and width and was isolated from the surrounding plots by 2 m. Onion bulbs were sown directly into the soil at a depth of 3 cm in four rows, with a row-to-row spacing of 0.45 m and a plant-to-plant spacing of 0.15 m. Plant density was similar across the plots. After sowing, all plots were irrigated individually.

### 2.3. Fertilization Treatment

Fertilizer treatments began six weeks after sowing, when plants reached an average height of 8–12 cm. Three levels of nitrogen (N) and phosphorus (P) fertilizer were applied in doses of 188 kg/hectare (T1, low), 375 kg/hectare (T2, moderate), and 750 kg/hectare (T3, high). Nitrophos fertilizer was applied three times, i.e., when plants reached a height of 8–12 cm, at 20% of flowering, and 60% of flowering. To estimate the flowering percentage, we counted the total number of flowering umbels at the anthesis stage, divided them by the total number of plants, and multiplied the result by 100.

### 2.4. Pollination Treatment

Four levels of insect pollination treatment were used to calculate the effectiveness of pollination, i.e., 0%, 25%, 50%, and 100%. Pollination treatments were assigned before the flower opening stage. Plants of similar height and vigor were randomly selected and assigned a pollination treatment level within each plot. Pollination gradients were managed by covering umbels with fine mesh net bags. We achieved 0% pollination by completely covering the umbels, 25% pollination by removing the fine mesh bags for one day and then covering them for three days, 50% pollination by removing the fine mesh bags for two days and covering them for two days, and 100% pollination by keeping the umbels uncovered. For all the treatments with the net bags, we adjusted the bags to avoid contact with the umbels [15]. We covered and uncovered the umbels between 10:00 am and 12:00 pm, which was the peak foraging time of the pollinators [10].

### 2.5. Yield Parameters and Pollinator Visitation Rate

At physiological maturity, umbels were manually harvested and placed in paper bags to prevent moisture accumulation, then dried for six weeks. Seeds were harvested by shaking the umbels. We calculated the umbel weight and seed number per umbel in grams.

During the flowering stage, pollinator diversity and abundance were recorded by randomly selecting ten umbels per plot and observing each umbel for one minute, counting the visiting insect pollinator species in each plot. The foraging behavior of abundant insect pollinators was recorded in terms of visit duration (time spent on an umbel) and the number of umbellules (flowers within an umbel) visited per visit (visitation rate). Observations were made between 10:00 a.m. and 12:00 p.m.

### 2.6. Effectiveness of Pollinators in Fertilizer Treatment

The effectiveness of pollinators was measured by recording pollen deposition from a single visit. Nine umbels of the same age, size, and vigor were visually selected from each fertilizer treatment plot. Umbels were covered with nylon mesh bags before flowering. During peak activity time (10:00 a.m.–12:00 p.m.) [10], the bags were removed when 50% of the flowers had opened. One pollinator (bee or fly) was allowed to visit the umbel, which was covered again after the pollinator visit [10]. Open-pollinated plants and caged plants (with no insect visitation) were also kept for yield comparison. Yield parameters, including umbel weight and number of seeds per umbel, were recorded using a digital weighing balance.

### 2.7. Statistical Analysis

Statistical analyses were performed using XL STAT and Statistics 8.1. Data for abundance, visit duration, and visitation rate of insect pollinators in each treatment were transformed using log transformation to ensure a normal distribution. After that, a two-way analysis of variance (ANOVA) was performed. When the interaction between factors was not significant, the main effects of each factor were evaluated independently within the two-way ANOVA. Similarly, yield parameters, i.e., umbel weight and seeds per umbel were analyzed. The means were separated using Tukey’s HSD all-pairwise comparisons test. The abundance, visit duration, and visitation rate of bees and flies were subjected to a paired-sample *t*-test.

## 3. Results

**Figure 1:**
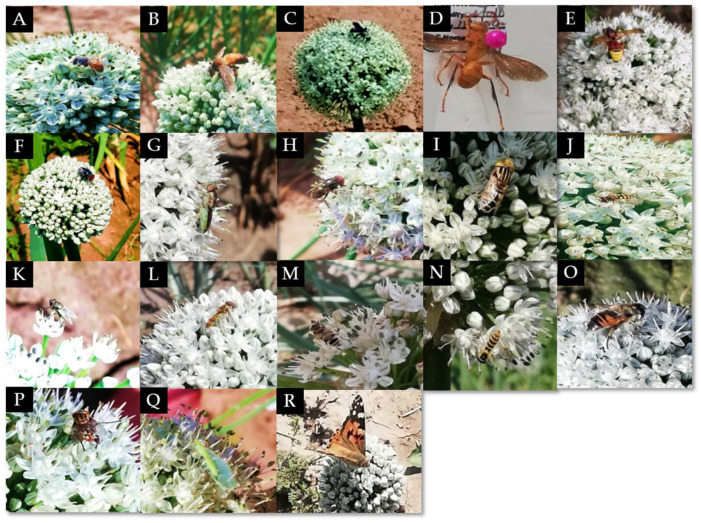
Different insect pollinators forage on onion crops: (**A**) *Apis florea*; (**B**) *Apis dorsata*; (**C**) *Xylocopa* sp.; (**D**) *Polistes* sp.; (**E**) *Vespa* sp.; (**F**) *Calliphora* sp.; (**G**) *Stratiomyidae* sp.; (**H**) *Eristalinus aeneus*; (**I**) *Eristalinus laetus*; (**J**) *Eupeodes corollae*; (**K**) *Musca domestica*; (**L**) *Episyrphus balteatus*; (**M**) *Eristalinus megacephalus*; (**N**) *Sphaerophoria bengalensis*; (**O**) *Eristalis tenax*; (**P**) *Sarcophaga* sp.; (**Q**) *Chrysoperla carnea*; and (**R**) *Vanessa cardui*.

The pollinators visiting onion umbels included three bee species, twelve dipteran fly species, and two wasp species (Figure 1). Among the bees observed, *A. florea* was the most abundant species, followed by *Xylocopa* sp. We also observed twelve dipteran fly species belonging to five families: Calliphoridae, Muscidae, Sarcophagidae, Syrphidae, and Stratiomyidae. The abundance of hoverfly *Eristalinus aeneus* was the highest, followed by *Episyrphus balteatus* and *Sphaerophoria bengalensis* (Table 1).

**Figure 2:**
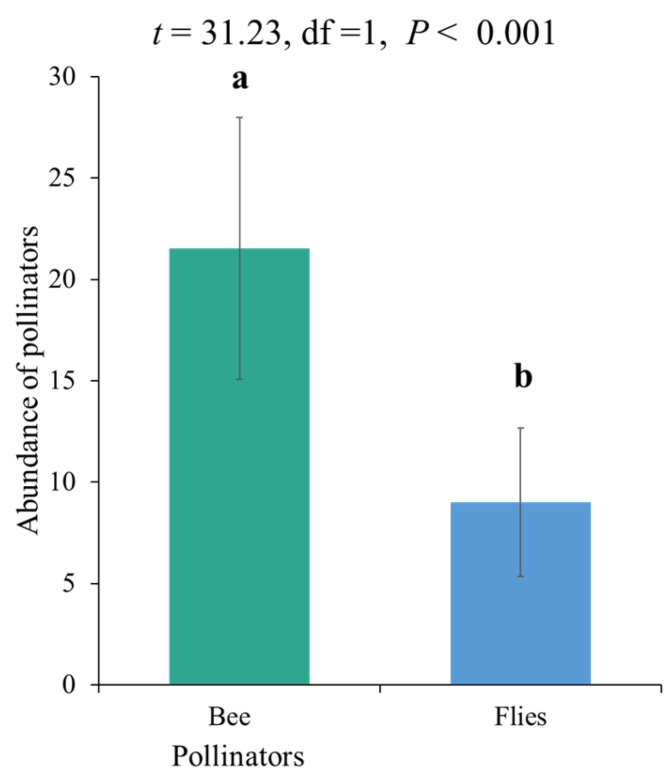
Overall abundance of onion crop pollinators (bees and flies) at all levels of fertilizer. Bars having different letters (a, b) show a statistically significant difference between groups based on a *t*-test (*p* < 0.05).

Other insects, such as *Chrysoperla carnea*, and a butterfly species, *Vanessa cardui*, were occasionally found visiting the onion umbels. No significant differences were found for the effect of fertilizer on the total abundance of major pollinator species, including bees and flies (*p* = 0.6784) (Table 2). Overall, the abundance of bees was significantly higher compared to flies (Figure 2).

**Figure 3:**
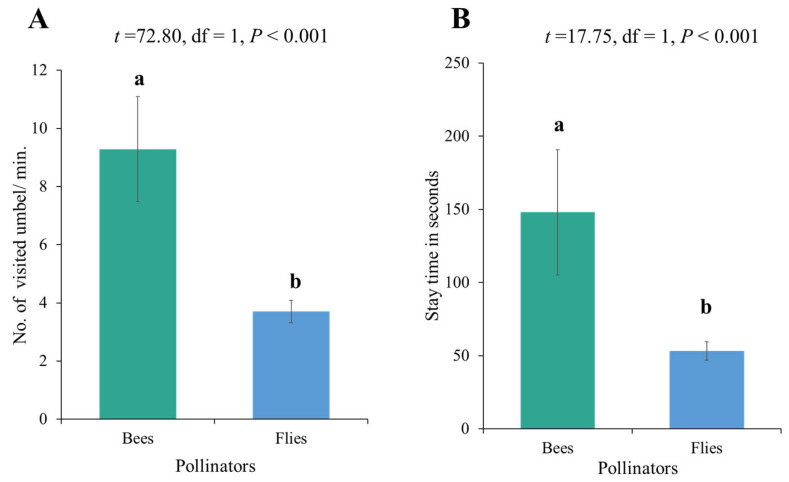
Foraging behavior of onion crop pollinators (bees and flies) at all levels of fertilizer: (**A**) no. of visited onion umbel/minute by bees and flies and (**B**) visit duration of bees and flies on the umbel. Bars having different letters (a, b) show a statistically significant difference between groups based on a *t*-test (*p* < 0.05).

**Figure 4:**
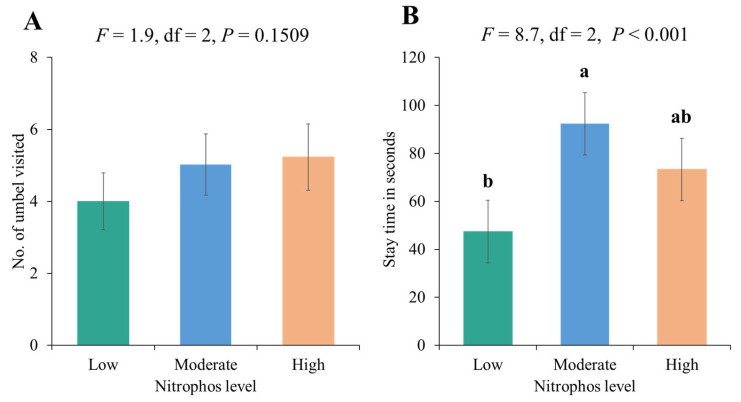
Foraging behavior of onion crop pollinators (bees and flies) at different levels of fertilizer application: (**A**) no. of visited onion umbel/minute by bees and flies and (**B**) visit duration of bees and flies on umbel. Bars with different letters are significantly different (Tukey’s HSD all-pairwise comparisons test). Bars without letters are not significantly different.

Overall, bees demonstrated a higher number of visits per umbel and a higher stay time per umbel compared to the flies (Figure 3). Furthermore, the visitation rate of primary insect pollinators on onions revealed differences across low-, medium-, and high-fertilizer plots. Bees had the highest visitation rate in medium-level fertilizer plots (T2), while flies had the highest visitation rate in high-level fertilizer plots (T3). The impact of fertilizer on the visit duration of bees and flies was not significant (*p* = 0.1509) (Table 3). Overall, moderate and high levels of nitrophos application led to significantly higher stay times of pollinators (bees and flies) compared to lower application levels. However, no significant differences were recorded for several umbels visited by pollinators (Figure 4).

The results of pollination treatments at low, medium, and high fertilizer levels showed that the maximum umbel weight and number of seeds were recorded in the 100% pollination treatment, followed by the 50% pollination treatment (Table 4). The highest umbel weight (8.04 ± 0.79) and number of seeds (45.22 ± 5.49) were observed at moderate fertilizer levels with the 100% pollination treatment. However, there was a decline in seed yield at high fertilizer levels (T3) with 100% pollination. The least reproductive success was recorded for 0% pollination across all fertilizer levels. The effects of fertilization and pollination treatments on onion umbel weight were insignificant (*p* = 0.567). Overall, the moderate fertilizer level (375 kg/hectare) combined with 100% pollination provided the highest seed yield in onions (Table 4).

**Figure 5:**
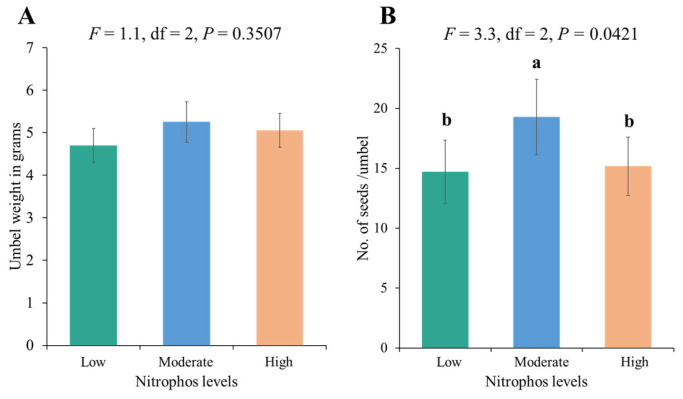
Yield parameters under three levels of fertilizer application: (**A**) weight of umbel in grams and (**B**) no. of seeds/umbel. Bars with different letters are significantly different (Tukey’s HSD all-pairwise comparisons test). Bars without letters are not significantly different.

**Figure 6:**
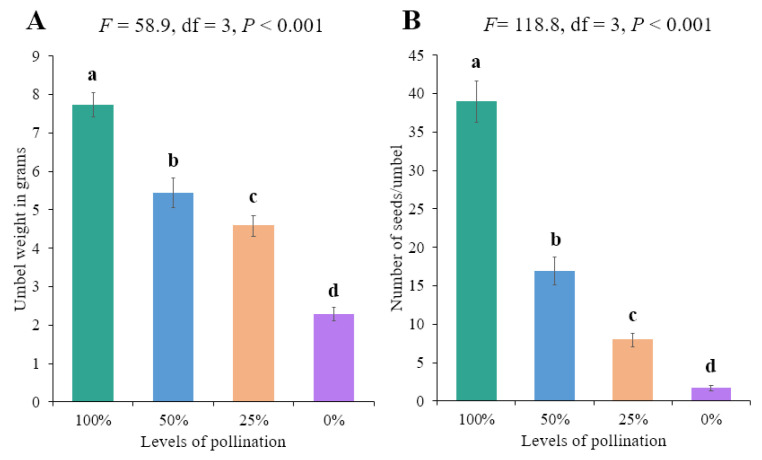
Reproductive parameters under different pollination treatments: (**A**) weight of umbel in grams and (**B**) no. of seeds/umbel. Bars with different letters are significantly different (Tukey’s HSD all-pairwise comparisons test).

The effectiveness of a single visit by bees and flies differed significantly in terms of umbel weight. Moreover, the effect on seed number was not significantly different (*p* = 0.615). The highest umbel weight was achieved with a single visit from bees at the low fertilizer level, followed by the high fertilizer level, with a slight difference (Table 5). Overall, a significantly higher number of seeds and a higher umbel weight were recorded for moderate nitrophos application (Figure 5). Moreover, the 100% pollination treatment led to higher umbel weight and number of seeds (Figure 6).

## 4. Discussion

The current study revealed that hymenopterans (71%) and dipterans (29%) were the most abundant visitors of onion flowers, while neuropterans and lepidopterans were occasionally found visiting the onion umbels. These findings aligned well with other studies that identified honey bees as the most abundant pollinators of onions [12,30,31,32]. Additionally, some studies have shown that syrphid flies played a vital role in onion pollination [10,33]. In our study, dipterans were the most species-rich order of onion pollinators.

Our research established the link between nitrophos fertilizer application and pollinator visitation. Hymenopterans (bees) preferred low- and medium-level fertilizer plots, while dipterans (flies) preferred to visit high-level fertilizer plots. The current results differed from the published findings, which showed that the bumble bee visitation rate was significantly higher for *Impatiens capensis* Meerb plants treated with high fertilizer amounts compared to those which had received low and no fertilizer treatments [34].

Published data showed that high fertilizer application altered the quantitative floral nutrition composition by changing the quality of nectar and pollen, which, in turn, influenced pollinator behavior [16,22,35,36,37]. It was shown that high fertilizer application decreased the quantity of essential amino acids in the plant and negatively affected bumble bees’ behavior [16]. The decreased visitation rate of onion flowers by bees in high-fertilization plots could be due to a similar effect of suboptimal fertilizer levels. The higher attraction of dipterans could be explained by their preferences for high levels of lipids, sugars, fructose, glycogen, and carbohydrates necessary for survival [38]. This finding suggests that high fertilizer levels can change the abundance and species composition of onion pollinators. On the other hand, another study reported that soil fertilizer application in okra, up to its optimal level, played an important role in improving the floral cues and attracting more pollinator fauna [1]. The impact of high fertilization levels on flower-visiting insects is pollinator-flowering plant species-specific and widely variable [39].

Our study showed that the benefits of pollination on onion yield could be maximized at moderate or recommended nitrophos applications, with a decrease in yield observed at higher nitrophos applications. Fertilizer application had a direct effect on the mature umbel weight [40]. We found that the combination of soil fertilization and insect pollination led to heavier umbel/plant in plants that were 100% pollinated compared to those with 0% pollination. Plants with 0% pollination, representing self-pollination, exhibited the lowest yield parameters due to suboptimal soil nutrients during both vegetative and reproductive growth phases, decreasing plant biomass production and yield parameters throughout the cropping seasons. This suggests that, while self-compatible plants can produce seeds without cross-pollination, they may experience reduced seed production in the absence of pollinators [10]. Our findings demonstrate that pollinators enhance onion seed production by increasing flower attractiveness and boosting seed production. We observed that exposure to 25% pollination had a minimal impact on onion yield, similar to when pollinators were entirely absent, while successful pollination exposures occurred at 50% and 100% under minimal and optimal soil fertilization. The variability in insect pollinator abundance suggests a potential influence on pollinator efficiency. The low yields in plants with 0% and 25% pollination were probably due to insufficient insect visitation, resulting in limited or no pollen transfer between plants, thus reducing the number and weight of seeds. This aligns with previous evidence, highlighting the positive interaction between soil fertilization and sufficient pollinators to improve sunflower and okra production parameters [1]. Another study reported that insect pollination enhances the average crop production by 18% to 71% (depending on the crop type) [41].

In addition, this study is the first example of single visits by insect pollinators, aiming to evaluate the effectiveness of pollinators at different nitrophos application levels. The mature umbel weight was highest where bees visited both at low and high levels of fertilizer, while a higher umbel weight was recorded at intermediate levels of fertilizer application. On the other hand, a decrease in umbel weight was seen when fertilizer levels increased. Interestingly, when the fertilizer was combined with a single pollinator visit, the amount of seeds produced per umbel was not significantly different. Furthermore, similar results were observed in fly single visits for all treatment levels, supporting the hypothesis that higher fertilizer concentrations reduce onion crop productivity and pollination efficiency.

Our results highlight the complex interlinkage between fertilizer application, crop productivity, and pollinator activity, emphasizing the importance of management strategies in agricultural landscapes. Understanding these mechanisms can help conserve the pollinator population. Further studies are needed to understand the physiological mechanisms associated with higher fertilizer application levels and low pollinator visits. Moreover, field trials should be conducted in different locations with different pollinator networks. Long-term studies are needed to evaluate the effects of different fertilizers on plant–pollinator interactions.

## 5. Conclusions

This study demonstrated the significant impact of nitrophos fertilizer application and insect pollinator visitation on onion (*Allium cepa* L.) seed production. Moderate fertilizer levels enhanced pollinator activity and onion umbel weight. Specific patterns of insect pollinator preferences emerged, with bees, including *Apis* species, favoring low-to-moderate fertilizer levels, while syrphid flies preferred higher levels. The highest umbel weight was achieved with the moderate fertilizer treatment, highlighting the bottom–up effects of moderate fertilizer use on pollinators and, consequently, onion seed yield. Future research could explore the use of other fertilizers, including micronutrients, and consider conducting long-term trials under diverse field conditions and across various crops to understand better fertilizer-mediated changes in pollinator behavior and their impact on crop yield.

## Figures and Tables

**Table 1:** List of insect pollinators foraging on onion flowers in plots with different levels of fertilizer.

Pollinator Group	Order	Family	Scientific Name
**Honey bees and wild bees**	Hymenoptera	Apidae	*Apis florea*
			*Apis dorsata*
			*Xylocopa* sp.
**Wasps**		Vespidae	*Vespa orientalis*
			*Polistes* sp.
**Flies**	Diptera	Calliphoridae	*Calliphoridae* sp.
		Muscidae	*Musca domestica*
		Sarcophagidae	*Sarcophaga* sp.
		Syrphidae	*Eristalinus aeneus*
			*Eupeodes corollae*
			*Sphaerophoria scripta*
			*Syrphus ribesii*
			*Episyrphus balteatus*
			*Eristalis tenax*
			*Mesembrius* sp.
			*Melanostoma* sp
		Stratiomyidae	*Hedriodiscus* sp.

**Table 2:** The abundance of major insect pollinators at different levels of nitrophos fertilizer.

Pollinator	Nitrophos Levels	Abundance
**Bees**	Low	14.72 ± 9.22
	Moderate	8.0 ± 5.09
	High	7.8 ± 5.2
**Flies**	Low	18.0 ± 10.21
	Moderate	22.25 ± 11.71
	High	24.33 ± 12.52
		*F* = 0.39, df = 2
		*p* = 0.6784

**Table 3:** Yield parameters under three levels of fertilizer application and different pollination treatments.

Pollination Treatment	Nitrophos Levels	Umbel Weight (Grams)	No. Seeds per Umbel
**100%**	Low	7.64 ± 0.73 a	37.00 ± 4.85
	Moderate	8.04 ± 0.79 a	45.22 ± 5.49
	High	7.50 ± 0.47 a	34.55 ± 2.88
**50%**	Low	4.68 ± 0.42 cd	13.55 ± 2.59
	Moderate	6.73 ± 0.67 ab	20.88 ± 2.18
	High	4.91 ± 0.74 cd	16.33 ± 3.94
**25%**	Low	3.98 ± 0.49 d	6.11 ± 1.24
	Moderate	4.29 ± 0.32 cd	9.77 ± 1.16
	High	5.44 ± 0.48 cd	8.00 ± 1.93
**0%**	Low	2.47 ± 0.29 e	2.11 ± 0.77
	Moderate	1.93 ± 0.26 e	1.22 ± 0.49
	High	2.36 ± 0.30 e	1.77 ± 0.68
		*F* = 2.13, df = 6	*F* = 0.81, df = 6
		*p* = 0.0567	*p* = 0.5677

Means within a column followed by different letters show significant differences, as determined by Tukey’s HSD all-pairwise comparisons test. The column without letters shows no differences between means.

**Table 4:** Visitation rate and visit duration of major pollinator groups on onion flowers at different nitrophos fertilizer levels.

Pollinator	Nitrophos Levels	Visitation Rate (No. of Visited Umbel/min.)	Visit Duration (Seconds)
**Bees**	Low	7.33 ± 2.52 b	93.37 ± 40.38
	Moderate	11.11 ± 3.59 a	203.86 ± 78.54
	High	9.44 ± 3.47 b	146.33 ± 96.33
**Flies**	Low	3.23 ± 0.75 c	36.77 ± 8.35
	Moderate	3.61 ± 0.44 bc	66.65 ± 11.61
	High	4.26 ± 0.76 bc	56.45 ± 11.71
		*F* = 8.68, df = 2	*F* = 1.91, df = 2
		*p* = 0.0002	*p* = 0.1509

Means within a column followed by different letters show significant differences, as determined by Tukey’s HSD all-pairwise comparisons test. The column without letters shows no differences between means.

**Table 5:** Single-visit efficacy at different levels of fertilizer.

Pollinators	Nitrophos Levels	Umbel Weight (Grams)	No. Seeds per Umbel
**Bees**	Low	9.27 ± 0.27 a	35.00 ± 0.82
	Moderate	6.98 ± 0.17 ab	32.00 ± 0.41
	High	7.45 ± 0.29 a	28.00 ± 1.08
**Flies**	Low	3.05 ± 0.28 c	16.60 ± 3.74
	Moderate	4.91 ± 0.81 bc	18.80 ± 4.20
	High	0.91 ± 0.48 c	16.80 ± 2.63
		*F* = 7.0, df = 2	*F* = 0.5, df = 2
		*p* = 0.0046	*p* = 0.6149

Means within a column followed by different letters show significant differences, as determined by Tukey’s HSD all-pairwise comparisons test. The column without letters shows no differences between means.

## Data Availability

The data used in this study can be provided by the corresponding author upon request.
